# Cardiometabolic Health Burden in Pediatric HIV: Unmet Need in the Contemporary Antiretroviral Therapy Era

**DOI:** 10.7759/cureus.85329

**Published:** 2025-06-04

**Authors:** Elisa López, Talía Sainz, Sahera Dirajlal-Fargo, Jennifer Jao, Jorge Pinto, Ann M Buchanan, Michael McKenna, Ana Milinkovic, Ana Puga

**Affiliations:** 1 Global Medical Affairs, ViiV Healthcare, London, GBR; 2 Pediatric Tropical and Infectious Diseases, Hospital Universitario La Paz and Hospital La Paz Institute for Health Research, Center for Biomedical Research in Infectious Diseases (CIBERINFEC) Universidad Autónoma de Madrid, Madrid, ESP; 3 Pediatrics, Northwestern University Feinberg School of Medicine, Chicago, USA; 4 Medicine, Federal University of Minas Gerais, Belo Horizonte, BRA; 5 Clinical Development, ViiV Healthcare, Durham, USA; 6 Safety, Evaluation, and Risk Management, GSK, London, GBR; 7 Global Pediatrics, ViiV Healthcare, Durham, USA

**Keywords:** adolescents, antiretroviral therapy, cardiovascular, children, hiv-1, metabolic health, pediatrics, weight

## Abstract

In children and adolescents with perinatally acquired HIV-1, early and prolonged exposure to HIV-1 and antiretroviral therapy (ART) can impact the premature development of cardiometabolic comorbidities. This narrative literature review aims to provide an overview of the impact of contemporary ART regimens on lipid profiles, body composition, and cardiovascular health in this population. Additionally, we propose recommendations for addressing cardiometabolic health in this understudied group. Studies show an increased risk of cardiovascular disease, adipose tissue dysfunction, and dyslipidemia among children and adolescents with perinatally acquired HIV-1. Clinical trial data support that contemporary ART regimens show better cardiometabolic profiles compared with older regimens. Emerging data suggest that chronic inflammation, epigenetic changes, gut barrier dysfunction, and oxidative stress are key mechanisms contributing to worsened cardiometabolic health in pediatric populations with HIV-1. People working with children and adolescents with HIV-1 should be aware of the increased risk of cardiometabolic health disorders in this population. More well-designed studies investigating cardiometabolic health in pediatric populations using contemporary ART regimens are needed. Early interventions during childhood and adolescence, critical developmental stages, may yield long-term benefits for health, quality of life, and longevity. A proactive, holistic approach to clinical management involving comprehensive care and early assessments of cardiometabolic health is essential to forestall the development of chronic disease.

## Introduction and background

Survival in the pediatric population with HIV-1 has improved substantially with antiretroviral therapy (ART) [1]. Children with HIV-1 transitioning into adolescence and adulthood represent a growing and important population [2,3] estimated at 3.2 million globally in 2022, with a majority living in low- and middle-income countries [4,5].

Cardiometabolic health has foundations in the fetal period into childhood and throughout the life course, and addressing cardiometabolic health early in development can prevent downstream chronic diseases and conditions [6-9]. As children with perinatally acquired HIV-1 age, cumulative exposure to HIV-1, ART, and associated inflammation can adversely influence cardiometabolic outcomes (Oral presentation: Dirajlal-Fargo S. Cardiometabolic Risks and Complications: Adolescents and Young Adults With Perinatally Acquired HIV. Conference on Retroviruses and Opportunistic Infections; 2024) [10]. Furthermore, adolescents with perinatally acquired HIV-1 and early-life exposure to older nucleoside reverse transcriptase inhibitors (NRTIs; particularly thymidine analogs, associated with mitochondrial toxicity) will have a higher risk of developing cardiometabolic health complications, as well as lipodystrophy, cardiomyopathy, liver toxicity, and dyslipidemia [2,5].

Here, we build on prior reviews on cardiometabolic health in children and adolescents with HIV-1 [2,5]. A hybrid approach was used to conduct a thorough, global, narrative literature review, combining a targeted literature review with expert recommendations from the authors. For the targeted literature review, we performed a series of literature searches using PubMed and Embase^®^ to identify contemporary studies and conference proceedings that were published or presented in any region worldwide. The initial search focused on articles and conference proceedings published or presented between January 1, 2011, and January 22, 2024, with relevance determined by title and abstract (see the appendix for the search terms). An additional PubMed search was performed to identify clinical trials involving pediatric and adolescent populations with HIV, published between January 1, 2011, and January 22, 2024. For this search, full-text articles were screened, and studies were included if they reported data on cardiometabolic health outcomes. To prioritize relevance to contemporary HIV treatment, only studies published in 2014 or later were considered. Studies involving young adults were included when deemed highly relevant or when both pediatric and young adult populations were included. To provide a more comprehensive overview, articles were also included based on the expert opinions of the authors, including articles or conference proceedings that were available through January 31, 2025. Key background articles that did not adhere to the above parameters were included to provide context (e.g., those explaining some cardiometabolic health endpoints).

## Review

Cardiovascular disease and surrogate endpoints

Understanding the risk of cardiovascular disease in children and adolescents with perinatally acquired HIV-1 is crucial. Clinical manifestations of cardiovascular disease are rare in this population [11]; therefore, surrogate endpoints are the usual assessment method for cardiovascular health.

The Pathobiological Determinants of Atherosclerosis in Youth (PDAY) score was developed using data from young adults aged 18 to 30 years without HIV-1 [12]. Across cohorts, including older children and adolescents with HIV-1 in the United Kingdom, United States, and South Africa, high proportions had PDAY scores ≥1, underscoring the elevated risk of developing atherosclerosis later in life [13-16]. For example, in a cohort of adolescents in the United Kingdom, 26% and 12%, respectively, had abdominal aorta and coronary artery PDAY scores ≥5 [13], and in the United States, 12% had coronary artery PDAY scores ≥4 [15]. Risk factors across studies for elevated PDAY scores were male sex, older age, viremia, and duration of ART use [14-16].

Structural and functional cardiac abnormalities, including right ventricular (RV) and left ventricular (LV) dilation, septal hypertrophy, and higher end-systolic wall stress, are a relatively common finding in children and adolescents with perinatally acquired HIV-1; the reported prevalence of cardiac conduction or functional abnormalities in this population is high and ranges from 14% to 42% (Table 1) [17-28].

**Table 1:** Summary of findings on cardiac complications in children, adolescents, and young adults with HIV-1. AA: abdominal aorta; ART: antiretroviral therapy; CA: coronary artery; CA-1: carbonic anhydrase 1; CCA-IMT: common carotid artery far wall intima-media thickness; cIMT: carotid intima-media thickness; CKD: chronic kidney disease; CRP: C-reactive protein; CT: computed tomography; EKG: electrocardiogram; EndoPAT: Endo Peripheral Arterial Tonometry; EN-RAGE: extracellular newly identified receptor for advanced glycation end products–binding protein; GDF-15: growth differentiation factor 15; IL: interleukin; IQR: interquartile range; LGE: late gadolinium enhancement; LPV: lopinavir; LV: left ventricular; MPI: myocardial performance index; MRI: magnetic resonance imaging; NVP: nevirapine; PDAY: Pathobiological Determinants of Atherosclerosis in Youth; PWV: pulse wave velocity; RV: right ventricular; ST-2: suppressor of tumorigenicity 2; TNF: tumor necrosis factor; VL: viral load; VSIG2: V-set and immunoglobulin domains–containing protein 2; ZDV: zidovudine. ^a^Percentages excluded if information not included in the source. ^b^Age range based on inclusion criteria. If inclusion criteria were not available, median age at baseline was reported. ^c^Virologic suppression rates reported if available. For prospective studies, baseline suppression rates were reported.

Author, year	Region	N with HIV-1 (% perinatally acquired)^a^	Age^b^	Design	ART use and virologic suppression^c^	Parameters	Findings
PDAY scores							
Greybe et al., 2024 [16]	South Africa	56 (100%)	Median, 17 y (IQR, 16-17)	Cross-sectional	100% on ART; 84% with VL <50 copies/mL	PDAY	59% and 55% of individuals had AA and CA risk scores, respectively, ≥1; older age was associated with an AA risk score ≥1
Karunaratne and Foster, 2017 [13]	UK	42	15-19 y	Retrospective	100% on ART; 86% with VL <50 copies/mL	PDAY	81% and 45% of individuals had AA and CA risk scores, respectively, ≥1; 26% and 12% had AA and CA risk scores, respectively, ≥5
Mahtab et al., 2024 [14]	South Africa	218 (100%)	9-14 y	Prospective	100% on ART; 38% with VL <50 copies/mL throughout the study	PDAY	30% and 18% of individuals had AA and CA risk scores, respectively, ≥1
Patel et al., 2014 [15]	US	165 (100%)	7-16 y	Prospective	87% on ART; 59% with VL ≤400 copies/mL	PDAY	48% had CA scores ≥1; 24%, 16%, and 12% had CA scores ≥2, ≥3, and ≥4, respectively; 24% had AA scores ≥1
Conduction and functional abnormalities							
Harrington et al., 2024 [26]	Kenya	176 (100%)	Children and young adults	Cross-sectional	100% on ART	Analysis of proteins, MPI	Individuals with abnormal MPI had higher BMI than those without (17.9 vs 16.7 kg/m^2^; P <0.001); 4 proteins were associated with MPI: VSIG2, CA1, EN-RAGE, and ST2; protein levels improved the discrimination of MPI cases in a clinical model
Githinji et al., 2019 [18]	South Africa	474 (100%)	9-14 y	Prospective	100% on ART; 78% with VL <50 copies/mL	Echocardiogram	RV dysfunction more common (32%) than LV dysfunction (7%) in individuals with HIV-1; no significant difference in the prevalence of RV or LV dysfunction between individuals with and without HIV-1
Lipshultz et al., 2017 [19]	US	214 (100%)	3-16 y	Prospective	35% on ART from 2004 to 2007; 65% naive to ART from 1990 to 1997	Echocardiogram	Individuals using ART had more normal LV function and structure than those naive to ART; cardiac function in individuals using ART decreased during 11 y of follow-up
Magodoro et al., 2023 [28]	South Africa	71 (100%)	14-19 y	Prospective	100% on ART; 62% with VL <40 copies/mL	Cardiovascular magnetic resonance	LV remodeling in adolescents with HIV-1 was associated with a distinct cardiovascular phenotype characterized by disproportionate expansion of the non-contractile interstitium without improved global circumferential strain
Majonga et al., 2018 [20]	Zimbabwe	197	6-16 y	Cross-sectional	100% on ART; 78% with VL <400 copies/mL	Echocardiogram	42% had an abnormality of either the left or right heart, with the most common (23%) abnormality being LV diastolic dysfunction; RV dilation and RV systolic dysfunction found in 7% and 2% of individuals, respectively
Majonga et al., 2020 [21]	Zimbabwe	175	6-16 y	Prospective	100% on ART; 78% with VL <400 copies/mL	Echocardiogram	At baseline, 23% had LV diastolic dysfunction, 18% had LV hypertrophy, 1% had RV systolic dysfunction, and 7% had RV dilation; over 18 mo, RV abnormalities, mean heart wall diameter, and tricuspid annular plane systolic excursion z-scores increased
Majonga et al., 2022 [22]	Zimbabwe	195 (100%)	6-16 y	Prospective	100% on ART; 78% with VL <400 copies/mL	Biomarkers, echocardiogram	Individuals with HIV-1 had higher levels of inflammation and cardiovascular biomarkers compared with those without HIV-1; increased levels of CRP and GDF-15 were associated with increased odds of LV abnormalities in individuals with HIV-1
McCrary et al., 2020 [23]	Kenya	643 (100%)	1-25 y	Cross-sectional	100% on ART; 77% with VL <1,000 copies/mL	Echocardiogram	Cardiac dysfunction in 28% of individuals; those with cardiac dysfunction were more likely to have ZDV exposure (P = 0.002); higher plasma IL-6 in those with cardiac dysfunction
Namuyonga et al., 2016 [24]	Uganda	285	1-18 y	Cross-sectional	100% on ART; 68% with VL <400 copies/mL	EKG, echocardiogram	14% had cardiac abnormalities; 5% had T-wave changes; 3% had pericardial disease
Wilkinson et al., 2018 [25]	US	246 (100%)	7-16 y	Cross-sectional	87% on ART; 69% with VL ≤400 copies/mL	Biomarkers, echocardiogram	Individuals with HIV-1 had higher biomarker levels (high-sensitivity cardiac troponin T, soluble TNF receptor II, IL-18) vs those without HIV-1; higher inflammation biomarkers generally associated with higher wall stress and lower LV mass and function
Williams et al., 2018 [27]	US	325 (100%)	7-16 y	Prospective	88% on ART; 69% with VL <400 copies/mL	Echocardiogram	LPV associated with better heart function scores
Williams et al., 2024 [29]	US	14 (100%)	Median, 29 y (IQR, 28-32)	Cross-sectional	100% on ART	Echocardiogram	Normal structure and systolic function observed in young adults with perinatally acquired HIV-1
Subclinical cardiovascular disease							
Abd-Elmoniem et al., 2014 [30]	US	35 (86%)	15-29 y	Cross-sectional	71% on ART; 43% with VL <50 copies/mL	CT, coronary vessel wall MRI	Right CA wall thickness was greater in individuals with HIV-1 vs controls (P = 0.002); smoking positively correlated with right CA wall thickness
Augustemak de Lima et al., 2018 [31]	Brazil	65 (100%)	8-15 y	Cross-sectional	83% on ART; 68% with VL <40 copies/mL	cIMT, biomarkers	Higher cIMT (P ≤ 0.03) in individuals with HIV-1 vs those without HIV-1; male gender, lower trunk fat, higher glucose, and IL-6 were associated with higher cIMT
Choudhury et al., 2021 [32]	Canada	90 (100%)	8-16 y	Cross-sectional	100% on ART; 100% with VL <40 copies/mL	Biomarkers	Individuals with HIV-1 had higher biomarkers of endothelial activation compared with reference ranges, possibly caused by gut barrier dysfunction, microbial translocation, and systemic inflammation; LPV/r use was associated with higher endothelial activation vs no use (P = 0.0020)
Davies et al., 2023 [33]	South Africa	141 (100%)	Median, 8 y (IQR, 8-9)	Prospective	100% on ART; 92% with undetectable VL	PWV	Children with HIV-1 on ART since infancy with excellent adherence had PWV that was not significantly different from that of children without HIV-1 (P = 0.080)
Dirajlal-Fargo et al., 2017 [34]	US	71 (25%)	12-28 y	Cross-sectional	100% on ART; 100% with VL <1,000 copies/mL	EndoPAT	Individuals with perinatally acquired HIV-1 had higher endothelial dysfunction vs those with other modes of HIV-1 acquisition and those without HIV-1 (P <0.01)
Dirajlal-Fargo et al., 2020 [35]	South Africa	283 (100%)	9-14 y	Prospective	100% on ART; 100% with VL <400 copies/mL	EndoPAT, biomarkers	Individuals with HIV-1 had worsened endothelial function compared with those without HIV-1 (P ≤ 0.001); individuals with HIV-1 had higher levels of soluble CD14; soluble CD14 correlated with endothelial dysfunction (ρ = 0.23; P = 0.007)
Dirajlal-Fargo et al., 2020 [36]	Uganda	101 (100%)	10-18 y	Cross-sectional	100% on ART; 100% with VL ≤400 copies/mL and 86% with <20 copies/mL	cIMT, PWV	cIMT was slightly thicker in individuals with HIV-1 vs those without HIV-1 (P <0.05); no significant difference observed for PWV (P = 0.06)
Dirajlal-Fargo et al., 2023 [3]	Uganda	101 (100%)	10-18 y	Prospective	100% on ART; 100% with VL ≤400 copies/mL and 86% with <20 copies/mL	cIMT, PWV	From baseline to 96 wk: median max cIMT decreased (P = 0.03) in individuals with HIV-1 and increased (P = 0.03) in those without HIV-1; PWV increased (P = 0.01) in individuals with HIV-1 but did not change significantly in those without HIV-1 (P = 0.92)
Dirajlal-Fargo et al., 2024 (Poster: Dirajlal-Fargo S, 2024)	Uganda	49 (100%)	10-18 y	Prospective	100% on ART; 100% with VL ≤400 copies/mL	PWV	Adverse childhood events were associated with higher PWV (P = 0.034); individuals with HIV-1 more likely to have higher adverse childhood event scores compared with those without HIV-1 (P = 0.015)
Hanna et al., 2016 [37]	US	221	6-29 y	Cross-sectional	48% on ART; 10% with VL <80 copies/mL	cIMT	In individuals with perinatally acquired HIV-1, CCA-IMT was non-significantly higher than those without HIV-1 (P = 0.08)
Innes et al., 2018 (Poster: Innes S, 2018)	South Africa	87	Median, 8 y	Prospective	100% on ART; 91% with VL <150 copies/mL	PWV	PWV was elevated in children with HIV-1 vs those without HIV-1 (P = 0.02); effect more pronounced in boys; PWV improved with accumulated time on ART
Jao et al., 2024 [38]	South Africa	365 (76%)	Median, 17-20 y	Cross-sectional	100% on ART; 39% with perinatally acquired HIV-1 with VL >50 copies/mL vs 13% with non-perinatally acquired HIV-1	Cardiovascular magnetic resonance	Median LGE mass and LGE fraction were significantly higher in individuals with HIV-1 vs those without HIV-1 (P ≤0.002)
Mahtab et al., 2020 [39]	South Africa	431 (100%)	9-14 y	Prospective	100% on ART; 62% with VL ≤50 copies/mL	EndoPAT	Individuals with HIV-1 had higher rates of endothelial dysfunction vs those without HIV-1 (50% vs 34%; P = 0.01)
Majonga et al., 2020 [40]	Sub-Saharan Africa	117 (100%)	6-16 y	Cross-sectional	100% on ART; 80% with VL <400 copies/mL	cIMT	cIMT did not significantly differ between individuals with HIV-1 vs those without HIV-1 (P >0.8)
Shiau et al., 2024 (Poster: Shiau S, 2024)	US	32 (100%)	Median, 11 and 17 y	Not reported	100% on ART	Biomarkers, epigenetics	Associations found between markers of inflammation and changes in gene expression involved in blood pressure regulation, CKD, and fatty acid metabolism
Urbina et al., 2025 [41]	US	95 (100%)	Median, 24 y (IQR, 21-26)	Cross-sectional	88% on ART; 82% with VL <400 copies/mL	PWV	Mean PWV was within standard ranges, although some individuals had PWV higher than the cutoff associated with cardiovascular events in adults
Williams et al., 2024 [29]	US	14 (100%)	Median, 29 y (IQR, 28-32)	Cross-sectional	100% on ART	Cardiovascular magnetic resonance	Perinatally acquired HIV-1 was not associated with diffuse myocardial fibrosis

Some evidence suggests a worsening of cardiac abnormalities in children and adolescents with HIV-1 using ART [19,21]. Cardiac function decreased over an 11-year follow-up in a prospective study in the United States [19]. Similarly, a prospective study in Zimbabwe reported a significant increase in RV dilation and systolic dysfunction and z-scores associated with heart wall diameter (P <0.05) through 18 months but no significant changes in left heart cardiac abnormalities [21].

In studies that used peripheral arterial tonometry to assess endothelial dysfunction, older children and adolescents with perinatally acquired HIV-1 who were virologically suppressed had increased endothelial dysfunction compared with those without HIV-1 (Table 1; participant ages ranged from 9 to 28 years, with two of three studies evaluating older children and adolescents only) [34,35,39]. Cardiac endothelial changes are an early indication of vessel wall alterations that occur before atherosclerosis [42].

Cardiac imaging-based findings generally support the risk of subclinical cardiovascular disease in adolescents and young adults with HIV-1 (Table 1) [29,30,38]. A cross-sectional study of individuals aged 15-29 years reported higher right coronary artery wall thickness among individuals with HIV-1 vs without HIV-1: mean (SD), 1.32 (0.21) vs 1.09 (0.14) mm; P = 0.002 [30]. An analysis of 464 adolescents and young adults (median age, 17-20 years) in South Africa assessed late gadolinium enhancement mass and fraction, an indicator of subclinical myocardial fibrosis [38]. In adjusted analyses, median late gadolinium enhancement mass and fraction were higher in youth with perinatally or non-perinatally acquired HIV-1 vs those without HIV-1. A pilot study assessed myocardial fibrosis in young adults with perinatally acquired HIV-1; the median (IQR) age was 29 (28-32) years [29]. Surprisingly, perinatally acquired HIV-1 was not associated with diffuse myocardial fibrosis in this study. The authors posited that early and sustained exposure to ART may have a role in mitigating cardiac fibrosis, but they also noted that the study population had a low prevalence of metabolic conditions and most had never smoked, factors that could have positively contributed to their cardiovascular health.

Carotid intima-media thickness (cIMT), a marker of cardiac structure, and pulse wave velocity (PWV), a marker of arterial stiffness, predict the likelihood of cardiovascular events [43,44]. cIMT and PWV were generally higher in studies of adolescents with HIV-1 (Table 1), indicating an increased risk of cardiovascular events as these individuals transition into adulthood (Poster: Innes S, Cotton MF, Otwombe K, Laughton B, Herbst PG, Karangwa I, Browne SH. Substantial Gender Differences in Longitudinal Arterial Stiffness in Children on ART. Conference on Retroviruses and Opportunistic Infections; 2018) [3,31,36]. However, the findings for PWV were not always consistent, and evidence supports that maintaining high ART adherence is associated with improved PWV (Poster: Innes S, 2018) [33,41].

Blood Pressure

High blood pressure is a key risk factor for cardiovascular disease, including heart failure and stroke [45]. There is conflicting evidence regarding the risk of high blood pressure in children and adolescents with HIV-1 (Table 2) (Oral presentation: Brady TM, Jacobson DL, Yu W, et al. Association of Perinatal HIV Infection With Blood Pressure and Hypertension Among Youth. Pediatric Academic Society Conference; 2022) [13,15,46-49]. For example, a retrospective study of children, adolescents, and young adults with HIV-1 in the United States reported that 20% had hypertension vs 3%-5% in the general pediatric population [46]. However, a separate analysis in the United States found that only 1% were hypertensive [15], and a more recent analysis found similar rates of hypertension between children and adolescents with HIV-1 and HIV-negative individuals exposed to HIV-1 (Oral presentation: Brady TM, 2022). High rates of blood pressure or hypertension were also observed in studies in the United Kingdom and Uganda [13,48]. In contrast, hypertension prevalence was low (0%-4%) in studies in Spain and South India [47,49]. Variability in findings may be due to differences in setting or exposure to risk factors such as ART composition. Individuals were using ART in most studies, although rates of virologic control varied, and few studies employed prospective designs. Hypertension was commonly defined as blood pressure ≥95th percentile.

**Table 2:** Summary of findings on blood pressure in children, adolescents, and young adults with HIV-1. ART: antiretroviral therapy; BP: blood pressure; HTN: hypertension; IQR: interquartile range; NA: not available; UK: United Kingdom; US: United States; VL: viral load. ^a^Age range based on inclusion criteria. If inclusion criteria were not available, mean or median age at baseline was reported. ^b^Virologic suppression rates reported if available. For prospective studies, baseline suppression rates were reported. ^c^Used a single BP measurement instead of the recommended multiple BP measurements.

Author, year	Region	N with HIV-1 (% with perinatally acquired)	Age^a^	Design	ART use and virologic suppression^b^	Definition of high BP or hypertension	Findings
Brady et al., 2022 (Oral presentation: Brady TM, 2022)	US	447 (100%)	7-16 y	Prospective	Proportion on ART not reported	NA	Systolic blood pressure and HTN rates were similar between individuals with HIV-1 compared with HIV-negative individuals exposed to HIV-1
Chatterton-Kirchmeier et al., 2015 [46]	US	266 (58%)	2-25 y	Retrospective	81% on ART; 65% with VL ≤400 copies/mL	High BP: ≥95th percentile in those aged <18 y; ≥140/≥90 mm Hg in those aged ≥18 y^c^	18% were categorized as having high BP; 20% of children and adolescents had high BP
Karunaratne and Foster, 2017 [13]	UK	42 (100%)	Median, 17 y (IQR, 16-18)	Retrospective	100% on ART; 86% with VL <50 copies/mL	HTN: ≥95th percentile	26% had HTN
Mehta et al., 2016 [47]	India	97 (100%)	3-18 y	Observational	Two-thirds on ART	HTN: ≥95th percentile; pre-HTN: ≥90th to <95th percentile	2% had pre-HTN; none had HTN
Migisha et al., 2023 [48]	Uganda	1,045 (52%)	13-25 y	Cross-sectional	100% on ART; 83% with VL <1,000 copies/mL	Elevated BP: 120/<80 to 129/<80 mm Hg; stage 1 HTN: 130/80 to 139/89 mm Hg; stage 2 HTN: ≥140/90 mm Hg	49% had high BP; 22% had elevated BP, 21% had stage 1 HTN, and 6% had stage 2 HTN
Patel et al., 2014 [15]	US	165 (100%)	7-16 y	Prospective	87% on ART; 59% with VL ≤400 copies/mL	HTN: ≥95th percentile at ≥3 clinic visits	1% had HTN
Sainz et al., 2014 [49]	Spain	64 (100%)	Mean, 14 y (SD, 5)	Prospective	100% on ART; 83% with VL <50 copies/mL	HTN: systolic BP >95th percentile	4% had HTN, which was similar to rates in individuals without HIV-1 (3%)

Associations Between ART and Cardiometabolic Health

Compared with untreated HIV-1, initiating ART improves cardiac structure and function [19]. However, data evaluating the association between contemporary ART and cardiovascular disease risk in pediatric populations are limited. Boosted protease inhibitors (PIs), especially first-generation PIs such as ritonavir-boosted lopinavir, have been associated with adverse lipid profiles, which contribute to cardiovascular disease [5]. Furthermore, ritonavir-boosted PIs were associated with endothelial activation [32], elevated PWV and cIMT (compared with nevirapine) [36], and higher PDAY scores. Surprisingly, one study reported that ritonavir-boosted lopinavir was associated with improved cardiac function as assessed by ejection fraction and fractional shortening [27]. As described in the lipids section below, newer antiretrovirals have a more favorable lipid profile and, thus, may have fewer adverse effects on the cardiovascular system.

In adults, abacavir has been associated with increased cardiovascular disease risk, though the association and potential underlying mechanisms remain controversial [50,51]. In a recent analysis of adults with HIV-1, current or former use of abacavir was associated with an ~42% to 50% higher risk of major adverse cardiovascular events (Poster: Fichtenbaum CJ, Malvestutto CD, Watanabe MG, et al. Abacavir Is Associated With Elevated Risk of Cardiovascular Events in the REPRIEVE Trial, International AIDS Conference; 2024). An analysis of adolescents found no significant association between current abacavir use and higher PDAY scores, whereas longer duration of boosted PI use and no prior tenofovir disoproxil fumarate (TDF) use were associated with elevated PDAY scores [15]. Additionally, another study demonstrated no association between abacavir use and cIMT in children and adolescents [52].

Biomarkers

Recent findings underscore associations between inflammation biomarkers and cardiovascular disease risk in children and adolescents with perinatally acquired HIV-1. Higher soluble CD14 positively correlated with increased endothelial dysfunction [35], and C-reactive protein and interleukin 6 were associated with changes in gene expression involved in blood pressure regulation, chronic kidney disease, and fatty acid metabolism (Poster: Shiau S, Brummel SS, Douglas J, et al. DNA Methylation Signatures of Inflammation in Youth Living With Perinatally-Acquired HIV. Conference on Retroviruses and Opportunistic Infections; 2024). Perinatally acquired HIV-1 may also disrupt the gut barrier and alter microbiota profiles and composition to drive chronic immune activation and subsequently may increase adiposity and risk of cardiometabolic complications [36,53].

Emerging data from proteomic studies, or the systematic study of protein expression, have implicated a set of proteins associated with cardiovascular disease in the prognosis of cardiovascular function in children and young adults [26]. Adding these protein levels into a clinical model improved the discrimination of positive cases with an abnormal myocardial performance index. Although further validation is needed, there may be potential for circulating proteins to provide insight into the prognosis of cardiovascular disease in this population.

Cardiovascular Disease Conclusions

Children and adolescents with perinatally acquired HIV-1 have an increased risk of cardiovascular disease. Specifically, this group is at risk of cardiac structure and function abnormalities [17-27] and often presents with signs of subclinical cardiovascular disease (Poster: Innes S, 2018) [3,28-31,36,38]. Generally high proportions of older children and adolescents have high PDAY scores [13-16] and elevated blood pressure, although these findings varied by setting (Oral presentation: Brady TM, 2022) [13,15,46-49]. Understanding the long-term clinical outcomes of these observations through the transition into adulthood is critical to reducing morbidity, improving quality of life, and promoting longevity.

Body composition and dyslipidemia

Understanding metabolic factors that contribute to cardiovascular disease in children and adolescents with HIV-1 is critical. We review two key metabolic disorders in the context of contemporary ART: body composition with a focus on weight gain and dyslipidemia.

Weight Gain

Obesity is a major cardiovascular disease risk factor [54,55]. The prevalence of people with overweight or obesity has increased globally, including among people with HIV-1 [50]. However, children and adolescents with HIV-1 can still experience growth deficits that begin early in life, especially in resource-limited settings [56]. Initiating ART improves growth, including weight [56]. HIV-related factors including untreated virus causing weight loss and potential weight gain after ART initiation (a “return to health”) make evaluating weight in this population complex as weight gain in a growing pediatric population is expected [57,58]. However, excess weight gain contributes to life-limiting chronic conditions and can impact quality of life, including self-perceived body appearance [50].

Clinical guidelines primarily recommend the second-generation integrase strand transfer inhibitors (INSTIs) dolutegravir or bictegravir as preferred core agents for first-line regimens in children and adolescents [59-61]. Contemporary ART regimens containing second-generation INSTIs and tenofovir alafenamide (TAF) have been associated with more pronounced weight gain in adults [50,57,62]. Thus, it is important to determine whether these regimens contribute to excess weight gain in pediatric populations.

Body mass index (BMI) z-score (BMIz) was the most frequently used method to assess weight. Most clinical trials were conducted in developing countries [63-67], whereas several non-interventional real-world studies were conducted in high-income settings (Poster: Foster C, 2023) [68-71]. Findings from clinical trials in children and adolescents show that weight gain after initiating second-generation INSTIs (primarily dolutegravir) is not excessive (Table 3 summarizes studies evaluating weight and contemporary ART) (Poster: McKenna M, Lulic Z, Buchanan AM, Brothers C, Tan L, Wang M, Brummel S, Ziemba L, Ruel T, Flynn PM, Rabie H, Wiznia A. Dolutegravir and Growth in Pediatric Populations With HIV-1: IMPAACT P1093 and IMPAACT 2019. Conference on Retroviruses and Opportunistic Infections; 2024) [63-67]. However, non-interventional studies reported inconsistent effects on excess weight gain with second-generation INSTIs (follow-up range: 12-26 months) (Poster: Foster C, Smith C, Henderson M, Cheung M, Fidler S. Metabolic Health and Bone Density in Youth Living With Perinatal HIV. Conference on Retroviruses and Opportunistic Infections; 2023; Poster: Crichton S, Edgar K, Scott K, et al. Changes in Body Mass Index Before and After Starting Dolutegravir and Compared to Protease Inhibitors in Children and Adolescents Living With HIV in Europe and Thailand. International AIDS Society Conference; 2024) [68-71].

**Table 3:** Summary of findings on ART and body composition in children, adolescents, and young adults with HIV-1. ABC: abacavir; ART: antiretroviral therapy; ATV: atazanavir; BIC: bictegravir; BMI: body mass index; BMIz: BMI z-score; CI: confidence interval; DRV: darunavir; DTG: dolutegravir; DXA: dual-energy radiograph absorptiometry; EFV: efavirenz; FTC: emtricitabine; INSTI: integrase strand transfer inhibitor; IQR: interquartile range; NNRTI: non-nucleoside reverse transcriptase inhibitor; NRTI: nucleoside reverse transcriptase inhibitor; PI: protease inhibitor; r: ritonavir; RAL: raltegravir; SD: standard deviation; SE: standard error; SOC: standard of care (predominantly NNRTI- or PI-based ART); TAF: tenofovir alafenamide; TDF: tenofovir disoproxil fumarate; 3TC: lamivudine; UK: United Kingdom; US: United States; WAZ: weight-for-age z-score; WHO: World Health Organization; ZDV: zidovudine. ^a^Percentages excluded if information not included in the source. ^b^Age range based on inclusion criteria. ^c^Reported based on available information in the source. ^d^796 of 833 participants with available data. ^e^Specific countries not available in the source.

Author, year	Region	N (% with perinatally acquired)^a^	Age^b^	Design^c^	Assessment	Findings
Integrase strand transfer inhibitors						
Amuge et al., 2022 [63]	Sub-Saharan Africa	85 (99%)	4 wk to <6 y	Randomized trial of DTG + two NRTIs vs SOC	BMIz	Comparable mean (SE) change in BMIz at 96 wk: DTG, 1.1 (0.3); SOC, 1.5 (0.3); P =0.50
Belfrage et al., 2023 [72]	Sweden	94	0 - 17 y	Retrospective cohort	BMIz	BMIz was not significantly different between DTG-based ART and ART without DTG
Crichton et al., 2024 (Poster: Crichton S, 2024)	Europe, Thailand	948 (96%)^d^	2 to <18 y	15 observational cohorts followed	BMIz	No statistical differences in mean BMIz over 96 wk with DTG vs PI-based ART (P =0.811); those aged 6 to <12 y had the most rapid increases in BMIz after initiating DTG; greatest increases in BMIz in those switching from EFV or TDF to DTG + TAF
Collins et al., 2019 [71]	UK, Ireland	304 (92%)	<18 y	Non-interventional study of initiating INSTI-based ART	BMIz	Change in median (IQR) BMIz at 12 mo: slight increases on RAL, 0.11 (−0.18 to 0.35); no change on DTG, 0.00 (−0.15 to 0.19)
Compagnucci et al., 2023 [64]	Uganda, South Africa, Ukraine, Thailand, Argentina, Mexico, Europe	318 (95%)	6-18 y	Randomized trial of switching to INSTI + DRV/r vs continuing SOC	BMIz	Increase in mean (SE) change in BMIz at 48 wk in INSTI + DRV/r: 0.23 (0.0) vs SOC: 0.01 (0.0); P <0.001; a low proportion of children had BMI ≥30 kg/mg^2^ at 48 wk (INSTI, 4/145; SOC, 3/153)
Foster et al., 2023 (Poster: Foster C, 2023)	UK	85 (100%)	15-24 y	Longitudinal study of INSTI vs SOC	BMI	No significant difference in change in BMI for INSTIs vs SOC at median follow-up of 26 mo
Frange et al., 2022 [68]	France	97	3-17 y	Retrospective, non-interventional study of DTG-based ART	BMIz	At 1 y post-DTG initiation, mean rate of change in BMIz was +0.08 (P = 0.10); change rate higher in adolescents vs children (0.16 vs −0.08; P =0.04)
Giacomet et al., 2021 [73]	Italy	13 (100%)	12-19 y	Non-interventional study of switching from SOC to DTG/ABC/3TC	DXA	Median (range) trunk fat increased from 23.9% (11.2%-33.7%) to 26.8% (11.9%-37.4%) at 12 mo (P =0.037)
Gaur et al., 2021 [67]	South Africa, Thailand, Uganda, US	100 (93%)	6 to <18 y	Single-arm trial of switching to BIC/FTC/TAF	WAZ	Median (IQR) change from baseline to 48 wk in WAZ was 0.27 (−0.01, 0.51)
Koay et al., 2021 [69]	US	38 (92%)	2-19 y	Retrospective study evaluating BMIz after initiating INSTI-based ART	BMIz	Adjusted difference in mean rate of change in BMIz increased post- vs pre-INSTI initiation (+0.19 z-score units/y; P =0.036)
McKenna et al., 2024 (Poster: McKenna M, 2024) (IMPAACT P1093)	Africa, Brazil, Thailand, US	181	4 wk to <18 y	Non-interventional single-arm trial of initiating DTG-based ART	BMIz	Overall mean (SD) BMIz increased from −0.1 (1.4) to 0.2 (1.3) at 48 wk
McKenna et al., 2024 (Poster: McKenna M, 2024) (IMPAACT 2019)	Africa, Thailand, US	57	6 mo to <12 y	Non-interventional single-arm trial of initiating DTG-based ART	BMIz	Overall mean (SD) BMIz increased from −0.7 (1.1) to −0.4 (1.2) at 48 wk
Mora et al., 2022 [74]	Italy	13 (100%)	12-18 y	Non-interventional study of switch to DTG-based ART	BMI, DXA	From baseline to 1 y, increases in mean BMI, 20.6 to 21.5 kg/m^2^; P =0.0134, and mean trunk fat, 20.2% to 23.1%; P =0.0276
Musiime et al., 2023 [65]	Uganda, Zambia, Zimbabwe	919	3-15 y	Randomized trial of initiating second-line ART	WAZ	Comparable increase in mean change in WAZ at 96 wk with DTG, DRV/r, or ATV/r
Thivalapill et al., 2021 [70]	Eswatini	460	10-19 y	Retrospective study of switch to DTG	BMIz	Individuals had decreasing BMIz/y (95% CI) before DTG transition, −0.1/y (−0.1 to −0.1) and increased BMIz/y, 0.3/y (0.2-0.3) post-initiation; difference, 0.4/y (0.3-0.5); P <0.001
Turkova et al., 2021 [66]	Europe, Asia, sub-Saharan Africa	707 (86%)	3-18 y	Randomized trial of DTG + two NRTIs vs SOC	BMIz	Minimal additional weight gain at 96 wk with DTG vs SOC (estimated between-group difference in mean BMIz, 0.13; 95% CI, 0.01-0.25); no significant increase in weight at 144 wk (estimated between-group difference in mean BMIz, 0.15; 95% CI, −0.08 to 0.37)
Tenofovir alafenamide						
Foster et al., 2023 (Poster: Foster C, 2023)	UK	85 (100%)	15-24 y	Longitudinal study of INSTI vs SOC	BMI	No difference in change from baseline BMI by ART including TAF, +0.6 (2.7) vs non-TAF, +1.4 (2.6); P =0.22 at median follow-up of 26 mo
Musiime et al., 2023 [65]	Uganda, Zambia, Zimbabwe	919	3-15 y	Randomized trial of initiating second-line ART	BMIz, WAZ	Significant increase in WAZ (P = 0.0001) and BMIz (P = 0.0005) for TAF + FTC vs ABC or ZDV + 3TC at 96 wk
Rakhmanina et al., 2020 [75]	International^e^	223	6 to <18 y	Analysis of four single-arm studies of suppressed switch to ART with TAF	BMI percentile	Weight gain through 48 wk was consistent with WHO data; younger age, lower baseline BMI percentage, and female sex associated with greater BMI percentile
Integrase strand transfer inhibitors plus tenofovir alafenamide						
Crichton et al., 2024 (Poster: Crichton S, 2024)	Europe, Thailand	948 (96%)^d^	2 to <18 y	Prospective study of 15 observational cohorts	BMIz	Switching from TDF or EFV to TAF + DTG was associated with the greatest weight gain trajectory through 48 wk
Musiime et al., 2023 [65]	Uganda, Zambia, Zimbabwe	919	3-15 y	Randomized trial of initiating second-line ART	WAZ	No evidence of excess weight gain with DTG and TAF (P =0.51) through 96 wk

The SMILE Penta-17-ANRS 152 trial assessed suppressed switch to an INSTI (97% on dolutegravir) + ritonavir-boosted darunavir vs continuing standard-of-care (SOC) non-NRTI (NNRTI)- or PI-based ART through 48 weeks (Table 3) [64]. This is the only contemporary pediatric trial evaluating a two-drug ART regimen without an NRTI backbone. Switch to INSTI + ritonavir-boosted darunavir was associated with a statistically significant increase in BMIz that was considered clinically modest; low and similar proportions of participants had BMI ≥ 30 kg/m^2^ in both groups. In the ODYSSEY study, initiating dolutegravir-based ART was not associated with excess weight gain through week 144 compared with SOC (NNRTI- or PI-based ART) [66]. Similar results were observed in subanalyses of participants naive to ART, participants switching to second-line ART, participants switching from TDF-containing regimens, and male and female participants [76]. In the IMPAACT 2019 and IMPAACT P1093 studies, children and adolescents initiating dolutegravir-based ART had overall small increases from baseline to week 48 in mean BMIz (Poster: McKenna M, 2024). More marked improvements in BMIz were observed among children and adolescents initiating dolutegravir-based ART with baseline BMIz < −2, supporting an impression of return to health in these individuals (Poster: McKenna M, 2024). In the CHAPAS-4 study of children and adolescents switching to second-line ART (median BMIz at baseline, −1.0), initiating dolutegravir-based ART was associated with similar weight gain to boosted atazanavir and darunavir, whereas boosted lopinavir was associated with no increase in weight.

In adolescents and adults, TAF is recommended in some preferred first-line regimens [59,61,77]. While randomized trials in adults have shown higher weight gain with TAF + dolutegravir vs TDF + dolutegravir or efavirenz [78,79], generally, these results have not been replicated in children and adolescents, though findings are inconsistent (Table 3) (Poster: Foster C, 2023; Poster: Crichton S, 2024) [65,75]. Two studies, including an analysis of four single-arm clinical trials, with follow-up periods ranging from 48 weeks to 26 months reported no excess weight gain associated with TAF use (Poster: Foster C, 2023) [75]. In contrast, the randomized CHAPAS-4 trial observed greater weight gain with TAF + emtricitabine vs abacavir or zidovudine + lamivudine over 96 weeks [65]. Findings regarding INSTIs + TAF were inconsistent (Poster: Crichton S, 2024) [65]. While the CHAPAS-4 study found no excess weight gain with dolutegravir + TAF [65], an analysis of children and adolescents in Europe and Thailand reported the greatest weight gain in individuals who switched from a regimen that included TDF or efavirenz to one that included both TAF and dolutegravir, although there were few individuals in this subanalysis (Poster: Crichton S, 2024). These discrepancies across studies may be due to differences in region, study design, comparator regimen, and prior efavirenz or TDF use.

Altered Body Composition

Body composition, which includes fat and lean mass, impacts multiple domains of cardiometabolic health [80]. Anthropometric changes, especially ectopic fat accumulation, are associated with metabolic disease [50]. Children and adolescents with perinatally acquired HIV-1 are at risk of adipose tissue dysfunction, which is associated with other metabolic disorders (Poster: Foster C, 2023) [52,81-86]. Some evidence supports that female children and adolescents with HIV-1 may have a greater likelihood of adipose tissue accumulation [83]. A longitudinal study found that they had greater increases in percent body fat and trunk fat compared with their male counterparts and with people without HIV-1. With contemporary ART, increased trunk fat has been reported one year after switching to dolutegravir-based ART in small observational cohorts of adolescents with HIV-1 [73,74].

Dyslipidemia

Atherogenic dyslipidemia, characterized by high levels of low-density lipoprotein cholesterol (LDL-C) and triglycerides and low levels of high-density lipoprotein cholesterol (HDL-C), is a major driver of cardiovascular disease risk [87]. Classically associated with obesity, insulin resistance, and type 2 diabetes, dyslipidemia is prevalent across different geographical settings and is associated with the use of ritonavir-boosted lopinavir compared with the use of NNRTI-based ART [31,49,88-91].

Switching from zidovudine or didanosine to TDF has been associated with improvements in lipid profile [92,93]. A longitudinal study reported an increase in total cholesterol in adolescents and young adults using TAF vs non-TAF ART but found no significant difference in triglycerides over a median follow-up of 26 months (Poster: Foster C, 2023).

Studies demonstrate a more favorable lipid profile with contemporary ART regimens, including second-generation INSTIs (Table 4) [63-66,94]. A prospective study reported that switching from older (primarily ritonavir-boosted lopinavir) to more contemporary PIs (ritonavir-boosted atazanavir or darunavir) was associated with an improved lipid profile after three years [94]. In the SMILE study, suppressed switch from NNRTI- or PI-based ART to INSTI + ritonavir-boosted darunavir was associated with similar total cholesterol, lower HDL-C, and a trend toward worsened LDL-C over 48 weeks [64]. The ODYSSEY and CHAPAS-4 trials reported favorable lipid profiles, with the exception of HDL-C (which decreased in ODYSSEY), with dolutegravir compared with NNRTI- or PI-based ART through 144 and 96 weeks of follow-up, respectively [63,65,66].

**Table 4:** Summary of findings on the effect of ART on lipids in children, adolescents, and young adults with HIV-1. ART: antiretroviral therapy; ATV: atazanavir; CI: confidence interval; DRV: darunavir; d4T: stavudine; DTG: dolutegravir; HDL-C: high-density lipoprotein cholesterol; INSTI: integrase strand transfer inhibitor; LDL-C: low-density lipoprotein cholesterol; LPV: lopinavir; NNRTI: non-nucleoside reverse transcriptase inhibitor; NRTI: nucleoside reverse transcriptase inhibitor; PI: protease inhibitor; r: ritonavir; SD: standard deviation; SOC: standard of care (predominantly NNRTI- or PI-based ART); TAF: tenofovir alafenamide; TDF: tenofovir disoproxil fumarate; UK: United Kingdom; US: United States; ZDV: zidovudine. ^a^Percentages excluded if information not included in the source. ^b^Age range based on inclusion criteria. ^c^Reported based on available information in the source.

Author, year	Region	N (% with perinatally acquired)^a^	Age^b^	Design^c^	Parameters	Findings
Integrase strand transfer inhibitors						
Amuge et al., 2022 [63]	Sub-Saharan Africa	85 (99%)	4 wk to <6 y	Randomized trial of DTG + two NRTIs vs SOC	HDL-C, LDL‑C, total cholesterol, triglycerides	Adjusted mean (95% CI) change from baseline to 96 wk for DTG vs SOC: non-significantly lower HDL-C, −3.0 (−10.3 to 4.3); significantly lower LDL‑C, −24.9 (−39.5 to −10.4); significantly lower total cholesterol, −24.4 (−40.3 to −8.5); no change in triglycerides, −9.0 (−39.6 to 21.7)
Compagnucci et al., 2023 [64]	Uganda, South Africa, Ukraine, Thailand, Argentina, Mexico, Europe	318 (95%)	6-18 y	Randomized trial of switching to INSTI + DRV/r or continuing SOC	Total cholesterol, LDL-C, HDL‑C	Adjusted difference (95% CI) in mean change from baseline to 48 wk for INSTI + DRV/r vs SOC: no change in total cholesterol, 0.3 (−5.7 to 6.4) mg/dL; P = 0.916; non-significant increase in LDL-C, 4.7 (−0.7 to 10.0) mg/dL; P = 0.088; decrease in HDL-C, −4.1 (−6.7 to −1.4) mg/dL; P = 0.003
Musiime et al., 2023 [65]	Uganda, Zambia, Zimbabwe	919	3-15 y	Randomized trial of initiating second-line ART	LDL-C, total cholesterol	Significant decrease in LDL-C and total cholesterol (P < 0.001) for DTG vs LPV/r
Turkova et al., 2021 [66]	Europe, Asia, sub-Saharan Africa	707 (86%)	3-18 y	Randomized trial of DTG + two NRTIs vs SOC	HDL-C, LDL‑C, total cholesterol, triglycerides	Adjusted mean (95% CI) change from baseline to 96 wk for DTG vs SOC: lower HDL‑C, −4.1 (−6.2 to −2.0); lower LDL‑C, −9.1 (−12.6 to −5.5); lower total cholesterol, −15.1 (−19.0 to −11.1); lower triglycerides, −21.5 (−28.9 to −14.1); similar effects were observed at 144 wk
Protease inhibitors						
Jao et al., 2018 [94]	US, Puerto Rico	167 (100%)	7-15 y	Prospective study of switch to ATV/r or DRV/r from an older PI	HDL-C, LDL‑C, total cholesterol, total cholesterol: HDL-C ratio, triglycerides	Switching to ATV/r or DRV/r was associated with a greater rate of decline in total cholesterol: HDL-C ratio (β, −0.12/y; P = 0.039) over time; greater decline in total cholesterol, 4.57 mg/dL/y (P = 0.057); no effect on HDL-C, LDL-C, or triglycerides
Tenofovir alafenamide						
Foster et al., 2023 (Poster: Foster C, 2023)	UK	85 (100%)	15-24 y	Longitudinal study of INSTI vs SOC	Total cholesterol, triglycerides	Mean (SD) total cholesterol higher for TAF vs non-TAF, 4.4 (0.9) vs 4.0 (1.0); P = 0.03 at median follow-up of 26 mo; no significant difference in triglycerides
Tenofovir disoproxil fumarate						
Aurpibul et al., 2015 [93]	Thailand	80	3-18 y	Prospective, non-randomized study of TDF vs non-TDF ART	LDL-C, total cholesterol	At 96 wk, TDF vs non-TDF had lower mean (SD) total cholesterol, 163 (26) vs 189 (35); P = 0.001 and LDL-C, 91 (23) vs 104 (31); P = 0.044
Saez-Llorens et al., 2015 [92]	Panama, UK, US	97	2 to <16 y	Randomized trial of switching to TDF vs continuing d4T or ZDV	HDL-C, LDL‑C, total cholesterol, triglycerides	TDF vs d4T or ZDV group had decreases in total cholesterol (median change, −14.0 vs 3.0 mg/dL; P = 0.005) and LDL-C (median change, −5.5 vs 6.0 mg/dL; P = 0.025) from baseline to 48 wk; no significant differences for HDL‑C or triglycerides

Biomarkers

Lipidomics, the large-scale study of lipid pathways and networks, allows for the characterization and quantification of lipid classes and species beyond that seen with traditional lipid panels that could help elucidate the underlying HIV- and/or ART choice-related mechanisms of dyslipidemia, especially in the setting of contemporary ART regimens [2,95]. In pediatric populations with HIV-1, altered levels of lipid species associated with cardiovascular disease risk and chronic inflammation, including diacylglycerols, cholesterol esters, ceramides, and saturated fatty acids, have been described [96-98]. Future studies could help ascertain the potential for lipidomic signatures as a biomarker of cardiovascular disease.

Body Composition and Dyslipidemia Conclusions

Overweight and obesity are important concerns and major risk factors for cardiovascular disease [54,55]. Findings from clinical trials support no excess weight gain with INSTIs (Poster: McKenna M, 2024) [63-67], providing reassurance that second-generation INSTIs are not a risk factor for developing obesity or overweight in pediatric populations. However, smaller observational studies have reported increased trunk fat with dolutegravir-based ART [73,74]. Given that altered body composition is a risk factor for cardiometabolic disease (Poster: Foster C, 2023) [52,81,82,84-86,99], longitudinal studies evaluating body composition are needed. Greater weight gain with INSTIs + TAF may occur after switching from efavirenz or TDF (Poster: Crichton S, 2024). This is consistent with evidence that second-generation INSTIs and TAF have weight-neutral effects and that more pronounced weight gain observed with these antiretrovirals in adults is caused by switching from antiretrovirals associated with weight suppression (primarily efavirenz and TDF) [57,62]. Furthermore, contemporary ART, including second-generation INSTIs, is associated with favorable lipid profiles compared with older regimens [63-66,94]. Emerging biomarker data reveal a role for lipidomics, chronic inflammation, and gut barrier dysfunction in cardiometabolic abnormalities (Poster: Shiau S, 2024) [31,32,35,96-98].

Recommendations

Children and adolescents with perinatally acquired HIV-1 continue to show evidence of subclinical cardiac and metabolic disease (e.g., increased risk of insulin resistance, dyslipidemia, subclinical cardiovascular disease, and liver complications) [2,5]. Cardiometabolic health in people with HIV-1, including children and adolescents, is multifactorial and influenced by lifestyle, genetics, individual characteristics, social determinants, HIV-1, and choice of ART [2,50,57,100-103]. Exposure to HIV-1 and ART in utero, lifelong ART use, long-term effects of older antiretrovirals associated with mitochondrial toxicity, and obesogenic environments influence cardiometabolic health risk in the pediatric population [2,104]. Ultimately, these factors can result in distinct clinical phenotypes compared with adults, including a metabolic paradox phenotype where individuals without obesity are metabolically unhealthy characterized by adipose tissue dysfunction, ectopic lipid accumulation, and insulin resistance, resulting in a higher risk of cardiometabolic disorders (Oral presentation: Dirajlal-Fargo S, 2024). Furthermore, children have different cardiometabolic risk factors and social determinants than those traditionally considered in adults (e.g., psychosocial stressors such as illness or death of a parent and rarely showing clinical manifestations of cardiometabolic disease) (Poster: Dirajlal-Fargo S, Shan S, Karungi C, et al. Influence of Childhood Adversity on Cardiovascular Health in HIV Youth in Uganda. Conference on Retroviruses and Opportunistic Infections; 2024) [105].

Building on prior work (Oral presentation: Dirajlal-Fargo S, 2024) [5,105] and drawing on the expertise of the authors of this paper, we propose recommendations for the scientific community, healthcare providers, and policymakers (Table 5). It is essential that people working with children, adolescents, and young adults with HIV-1 are aware of the increased risk of cardiometabolic disease in this population. Research recommendations highlight the need for longitudinal studies employing a holistic approach to evaluate cardiometabolic health to delineate the long-term trajectories and potential clinical implications as children and adolescents with perinatally acquired HIV-1 transition into adulthood. Research into the pathogenesis of cardiometabolic dysfunction can inform future treatment strategies. Childhood and adolescence are crucial windows for diet and lifestyle modification, presenting opportunities for prevention [6]. Pharmaceutical interventions with the potential to improve cardiometabolic health are also of particular interest. In a randomized phase 3 study, pitavastatin was associated with a lower incidence of major adverse cardiovascular events in adults with HIV-1, prompting an update to US treatment guidelines [61,106]. There is a need for guidance on statin use in younger populations with HIV-1 given their increased risk of cardiovascular disease.

**Table 5:** Key research and policy and clinical recommendations to advance the management of cardiometabolic health in children, adolescents, and adults with HIV-1. ART: antiretroviral therapy.

Research recommendations
Well-designed studies controlled for confounders are needed to assess the following:
Epidemiology including incidence and prevalence of cardiovascular disease and metabolic risk factors among individuals using contemporary ART regimens
Long-term trajectories and clinical implications of changes in cardiometabolic health as children and adolescents with HIV-1 transition into adulthood
Pathogenesis of cardiometabolic disorders
Interventions including lifestyle, use of statins, and reduction in inflammation
New scalable tools tailored to children and adolescents with HIV-1 are needed to assist in decision-making for evaluation, diagnosis, and treatment of cardiometabolic health disorders
Studies should use a holistic approach to the investigation of cardiometabolic health
Settings with the highest burden of HIV-1 should be considered when designing studies of cardiometabolic health
Policy and clinical recommendations
Healthcare providers and policymakers should be aware of the increased burden of cardiometabolic disease in children and adolescents with HIV-1
In addition to improving the lives of people with HIV-1, addressing cardiometabolic disease will likely reduce financial costs
Preventing cardiometabolic disorders will reduce downstream burden on the healthcare system
Addressing cardiometabolic health could improve quality of life and longevity, allowing for improved workforce engagement
An important goal for clinical management is minimizing morbidities throughout the life stages of growth and development
There is a need for standardization of metabolic parameters to study and assess cardiometabolic health
Diagnosis and management of cardiometabolic health is a key component of comprehensive care
Healthcare providers should be proactive in screening and monitoring cardiometabolic health parameters
Early screening and interventions and long-term monitoring of cardiometabolic health are critical during growth and development in the pediatric population with HIV-1
Blood pressure should be regularly monitored in older children and adolescents with HIV-1
Diagnosis and management should take a holistic approach including considerations of choice of ART, such as prior exposure to older antiretrovirals associated with mitochondrial toxicity and lipid elevations, family history, genetics, and an individual’s environment

The prevention of chronic disease in children and adolescents with perinatally acquired HIV-1 is of the utmost importance and requires addressing unmet needs surrounding cardiometabolic health. There is a need for clear guidelines and consensus definitions of cardiometabolic endpoints tailored to this population as well as validated diagnostic tools. Guidelines and diagnostic tools must consider scalability and expedience for implementation into overstretched healthcare systems [105].

The strengths of our review include thorough data on cardiovascular disease and the impact of contemporary ART on weight and lipid profiles. The scope of future reviews could extend to other critical aspects of cardiometabolic health, including insulin resistance, diabetes, and liver health, which are discussed in detail in other reviews [2,5].

## Conclusions

Children and adolescents with HIV-1 have an increased risk of cardiovascular disease early in life, driven in part by chronic HIV-related inflammation and exposure to ART. Overweight, obesity, and dyslipidemia are key contributors to this risk, underscoring the need for prevention and management. Contemporary ART regimens have shown better cardiometabolic profiles compared with older regimens.

Given these evolving insights, we emphasize the importance of early awareness, monitoring, and intervention to mitigate risks to cardiometabolic health, forestall chronic disease, and ultimately optimize long-term health outcomes in this important population. Clear guidelines, consensus definitions of cardiometabolic endpoints, and validated diagnostic tools designed for scalability and feasibility are essential. Interventions must be guided by well-designed studies evaluating pharmacological and lifestyle interventions tailored toward this population with considerable unmet needs.

## Appendices

Targeted literature review search strings

*Search*: Pediatric metabolic health via PubMed

*String*: (HIV[Title/Abstract]) AND (Pediatric* OR child* OR adolescent or "young adults" or neonate* or infant*) AND (prep OR "preexposure prophylaxis" OR "integrase inhibitors" OR raltegravir OR elvitegravir OR dolutegravir OR cabotegravir OR tenofovir OR TAF OR TDF OR NRTI*) AND (hypertension OR "high blood pressure" OR BMI OR "metabolic syndrome" OR diabet* or prediabetes "HbA1c" OR lipid* OR "insulin resistance" OR "HOMA-IR" OR glucose OR cardiac OR weight OR Fib-4 OR "hepatic steatosis" or "hepatic fibrosis" OR NAFLD OR height OR growth OR BAZ OR WAZ OR atherosclerosis OR vascular OR "carotid artery")

*Search*: Pediatric clinical trials via PubMed

*String*: (HIV[Title/Abstract]) AND ((Pediatric*[Title/Abstract] OR child*[Title/Abstract]) OR (adolescents[Title/Abstract]) or ("young adults"[Title/Abstract]) or (neonate*[Title/Abstract]) or (infant*[Title/Abstract])) AND (prep OR "preexposure prophylaxis" or "integrase inhibitor*" or INSTI* or raltegravir or elvitegravir or bictegravir or dolutegravir or cabotegravir or tenofovir or TDF or TAF or NRTI*) AND ("clinical trial" or "phase 3" OR "Phase III" or "phase 2" or "randomized" or "open-label" or "phase II" or "phase 2/3") AND ("safety" or "metabolic" or cardiometabolic or cardiac or cardio* or insulin or lipids or glucose or cholesterol)

*Search*: Pediatric metabolic health via Embase^®^

*String*: hiv:ti,ab,kw AND (pediatrics:ti,ab,kw OR child:ti,ab,kw OR adolescent:ti,ab,kw OR neonate:ti,ab,kw OR young:ti,ab,kw OR infant:ti,ab,kw) AND (hypertension:ti,ab,kw OR 'high blood pressure':ti,ab,kw OR bmi:ti,ab,kw OR 'metabolic syndrome':ti,ab,kw OR cardiovascular:ti,ab,kw OR diabet*:ti,ab,kw OR prediabetes:ti,ab,kw OR hba1c:ti,ab,kw OR lipid*:ti,ab,kw OR 'insulin resistance':ti,ab,kw OR 'homa ir':ti,ab,kw OR glucose:ti,ab,kw OR cardiac:ti,ab,kw OR weight:ti,ab,kw OR 'fib 4':ti,ab,kw OR 'hepatic steatosis':ti,ab,kw OR 'hepatic fibrosis':ti,ab,kw OR 'nonalcoholic fatty liver':ti,ab,kw OR height:ti,ab,kw OR growth:ti,ab,kw OR baz:ti,ab,kw OR waz:ti,ab,kw OR atherosclerosis:ti,ab,kw OR vascular:ti,ab,kw OR 'carotid artery':ti,ab,kw)
